# Unbuilding the city: Deconstruction and the circular economy in Vancouver

**DOI:** 10.1177/0308518X221116891

**Published:** 2022-07-27

**Authors:** Nicholas Lynch

**Affiliations:** Department of Geography, Faculty of Humanities & Social Science, 7512 Memorial University of Newfoundland, Canada

**Keywords:** Circular economy, construction, renovation and demolition (CR&D), waste, deconstruction, Vancouver

## Abstract

Globally, the construction, renovation, and demolition sectors are increasingly responsible for growing resource demand and structural waste, even given progress in energy efficient technologies, ‘green’ building design, and local planning regulations. In response, the Circular Economy has become a popular agenda in the construction, renovation, and demolition sector as it offers a new model that not only maximizes materials reuse and recovery but also reframes urban systems and the built environment in a closed-loop (cradle-to-cradle) paradigm. In particular, popular visions of the Circular Economy promote, among other actions, ‘optimizing’ the end-of-the-life of buildings and their materials. Deconstruction (i.e. piece-by-piece demolition) is one key optimization strategy that has received increasing, yet limited, attention by researchers. This paper traces the development of an incipient deconstruction sector in Vancouver, focusing on the possibilities and challenges of deconstruction and material recovery practices as viable strategies for a transformative Circular Economy. I investigate two related aspects: first, the emerging policy landscape surrounding green demolition, and second, the development of ‘unbuilding’ practices and more formal ‘Deconstruction Hubs’. Overall, the paper finds that while these developments represent fundamental steps towards a more sustainable built environment, there remain a number of significant social, political and economic limitations that must be confronted if we are to meet the growing demands for more radical sustainability and ‘circularity’ not only in Canadian construction, renovation, and demolition sectors, but across Canadian cities and beyond.

## Introduction

[B]uilt environments are always at the same time unbuilding environments… (Cairns and Jacobs, 2014: 197)

Cities and their buildings have life-histories: they are conceived, develop, grow, transform, and even ‘die’. Architects, designers and developers, however, are habitually concerned with the creative acts of building, and spend much less attention to ‘the negative, anxiety-inducing flip side… [d]eath, destruction, and deterioration’ (Cairns and Jacobs, 2014: 1). In response, scholars from across the sciences and social sciences have increasingly drawn attention to the complex ‘end’ phases of buildings’ life cycles – from the processes of obsolescence (Abramson, 2016), abandonment and ruin (Edensor, 2005), the socio-politics of demolition (Akers et al. 2020), and finally, to the prospects of reuse – in whole or in part (Lynch, 2022). This paper focuses on the latter phases, demolition and reuse, to explore alternatives to the seemingly endless piles of rubble, debris and waste.

Globally, the construction, renovation, and demolition (CR&D) sectors, those vast industries of builders, remodelers and demolitionists, are part of a practice that generates mountains of structural waste, from the production of material discards during construction practices to the accumulation of enormous quantities of renovation and demolition materials that are increasingly landfilled (Wu et al., 2014). To be sure, sustainability strategies such as the integration of energy efficient technologies, ‘green’ building design and planning policies that improve livability have made gains over the years. The global uptake of green building strategies, for instance, of which LEED standards are perhaps the most popular, has rapidly transformed the design and construction sectors (Cidell, 2009). While important, however, such initiatives are not standard practice and prioritize the use phases (e.g. design and operation) of a building’s life and thus tend to ignore the sustainability challenges at ‘the end of pipe’. Considering this gap there have been increasing calls from academics for a deeper recognition and intervention in other, less explored, aspects of building lifecycles and the wider social, political and economic landscapes in which they are embedded. While some call for technical changes in CR&D production practices, others are arguing for more radical and holistic changes that transform the cultures of production, consumption and discard.

The Circular Economy (CE) is one agenda that has received increasing attention of late, particularly given its system-wide approach to rethinking the built environment in a more transformative way (EMF, 2015; 2020). In the last few years, the CE has expanded from its roots in Industrial Symbiosis, a well-developed field of resource and energy exchange, to become a central policy, planning and technical strategy in achieving resource efficiency and societal sustainability. Here, the contemporary CE is most commonly understood as an agenda that replaces traditional linear approaches to consumer goods (e.g. take-make-waste) with products and assets that are inherently durable and repairable, and available through practices of refurbishment, reuse and disassembly. More recently, a key target for leading CE proponents is the urban environment and its resource intensive CR&D sectors. A formative part of this strategy involves recognizing and intervening in the process of urbanization in several ways including promoting platform economies that optimize building utilization and enabling technological changes that encourage material efficiency. Such strategies have gained considerable popularity (and critique) in the last few years, especially since they reflect a prioritization of technological solutions to meet growing ecological challenges during the early phases of a building’s lifecycle (EMF, 2015; 2020). Other significant practices, like deconstruction, that focus on the end of the life cycle have received less attention. Deconstruction is the piece-by-piece, and often hand-by-hand, dismantling of buildings as a means of recovering valuable materials (e.g. brick, wood, architectural elements). A longstanding but narrowly applied practice, deconstruction is a much slower but more nuanced, methodical, and skilled approach when compared to conventional mechanical demolition. While gaining some traction in Europe, deconstruction remains a relatively marginal practice in the CR&D sectors across North America, especially as traditional demolition continues to offer rapid cost-effective removal of the built environment (Creba and Devileger, 2019). In keeping up with the mounting pressures for urban (re)development, however, it is increasingly clear that conventional demolition and the landfilling of demolition debris is not a sustainable solution. In recent years, CE practitioners and a select number of cities in the US and Canada, notably Portland and Vancouver, are responding to such challenges through the incorporation of CE thinking in the building sectors and in the experimentation of deconstruction as alternatives to conventional demolition practices. Remarkably, however, deconstruction has received only limited attention in the social science literature. Little is known about the various possibilities and challenges presented by deconstruction in specific urban regions and its relationships to the emerging CE agenda in North America. This is especially true in Canada as the CE is just now receiving more widespread national attention and where cities, large and small, are grappling with alternatives to landfilling CR&D wastes. Given these developments, vital questions remain: How are local urban and industry leaders integrating, negotiating, and implementing circular practices in regional and municipal contexts? How viable, scalable, and effective are circular practices, like deconstruction, in the built environment sector?

With these questions in mind, this paper investigates deconstruction in Vancouver, focusing on the social, cultural and material politics of delivering the practice as a part of a regional CE. Through analyses of interviews with key informants and recent policy documentation, this work contributes to ongoing debates in urban studies and other critical social sciences that critique the CE and material reuse in the development of the so-called ‘Circular City’(CC) (Bassens et al. 2020; Kębłowski et al. 2020; Prendeville et al. 2018). I argue that the development of the deconstruction sector, increasingly framed as a circular practice, is inherently tied to local politics of green growth and nascent, sometimes conflicting, cultures of the CE. Thus, beyond underlining the main policies, programs and innovative approaches that are working to develop a meaningful deconstruction and material reuse sector in Vancouver, this paper highlights the important and complicated role of ‘unbuilding’, alongside the practice of building, the Circular City.

### Circular urbanism: critical geographies of the circular city

Over the last few years, the deepening relationship between the CE and the ‘urban’ has become readily apparent (Bassens et al., 2020; Keblowski et al., 2020; Savini, 2019; Williams, 2021). In road maps, reports, and project guides from leading CE organizations, global corporations and regional governments, the message is clear: ‘our cities of the future will be circular’ (EMF, 2020; WEF, 2018). Urban policymakers, in particular, have increasingly latched on to visions of circularity that place their cities as both actors and beneficiaries of circularity. Indeed, the CE has become a leading model of urban (re)development that encourages closed loop actions through a variety of circular practices: from industrial symbiosis and platform economies (e.g. TaskRabbit, Uber) to deconstruction. With these and other tactics in mind, a rapidly growing volume of research and marketing materials are framing cities, across Europe, China and now North America, as spaces of circularity, ‘key entities apparently solving the contradiction between scarce resources and unlimited growth’ (Bassens et al., 2020: 894). In short, ‘the CE has come to town’ (Keblowski et al., 2020: 142). And yet, as cities experiment with a dizzying variety of circular strategies in an endeavor to proclaim Circular City (CC) status, much remains unclear as to how this practice plays out in the city, that is, how the CE is developed and deployed, experimented, and challenged at the urban scale.

Some of these crucial questions are the focus of recent scholarship in geography and other critical social science disciplines. One starting point concerns the ongoing debates about the roots, definitions, and principles of the contemporary CE (Kirchherr et al., 2017). From the outset, CE research has been, and continues to be, led by the physical sciences and, more recently, by regional policy experts and corporate consultants. Although crucial in developing industrial applications and innovative service systems, there are rising concerns that the prioritization of these approaches construct and, indeed, affirm rather ‘potent and reassuring discourses of a sustainable future’ (Hobson, 2016: 3). Several recent critiques, for instance, highlight that such discourses are powerful technical narratives that uncritically ‘solve’ the inherent challenges of economic growth and resource use. Importantly, these narratives, which typically showcase digital infrastructure (e.g. Smart cities, big data, apps/platforms) and ‘green’ technologies, are ‘endlessly recited ideals’ (Gregson et al., 2015: 218) of circularity featured in policy and consultant reports that travel globally across socio-spatial contexts. For Hobson and Lynch (2016: 17), the prevailing CE, deployed in recent EU policy frameworks and championed by CE intermediaries like the EMF, mirror contemporary ecological modernist arguments, that ‘through technological and policy innovation, we can overcome environmental crisis without leaving the path of modernization’. The key point here is that a focus on tweaks, nudges and incremental reformations of ‘capitalism’s technical components’ leads less to radical change and instead affirms a logic of ‘sustainability-as-usual’, one that prioritizes economic considerations (e.g. profit and growth) (Godelnik, 2021).

For urban scholars, the implications of these ‘elegant’, ‘conciliatory’, and arguably superficial, discourses of the CE present considerable challenges, especially given growing concerns around urban inequality, resource (in)security, and the role of urban systems in waste accumulation and climate change (Bassens et al. 2020: 894; Keblowski et al.*,* 2020: 145). Along these lines, scholars question the vague use of circular language and tactics by city managers and consultants in adapting local (sustainability) policy (Prendeville et al. 2018). With loose definitions of the CE, urban agendas are vulnerable to a type of ‘circle washing’, where urban leaders can make circular claims without implementing real (i.e. radical) change (Valenzuela and Bohm, 2017). This is, of course, more than a mere branding exercise, as such tactics ‘indicate the pressure to frame the CE as one of ready-made “best practices”, of competition-driven, “mobile urbanism”’ (Bassens et al., 2020: 894). Over the last few years, the rapid diffusion of CC labels, from Amsterdam to Toronto (circulartoronto.com), speaks to the remarkable mobility and adaptability of circular strategies across urban space. Reflecting ‘fast policy’ (Peck and Theodore, 2015) inherent in contemporary neoliberal urbanism, the prevailing CE agenda thus points to yet another iteration of the ‘sustainability fix’, a growth-oriented discourse that supports urban development by accommodating both environmental concerns and profit making (While et al., 2004). Rather than forging the steps to a ‘future proof’ global society, circular cities may indeed amplify the punitive consequences of neoliberal urbanism, including exacerbating urban social and spatial divisions and prioritizing ‘the installation of technocratic governance structures evacuated from citizen participation’ (Keblowski et al. 2020:143). In terms of the latter, geographers have highlighted how CE and CC agendas too often ignore or disembed the role and practices of local consumers and urban citizens (Hobson, 2020; Myland et al. 2016). Recent work points out that while most CE research and strategy focus on structural and functional shifts (e.g. manufacturing, design, policy), our underlying understanding of how citizens consume, how they ‘take-up’, express, or indeed, challenge, circular practices in the spaces of the city is left underexplored (Hobson, 2020).

Last, and related, recent urban scholarship has questioned the wider implications of urban CE agendas as strategies that usher in new expressions of technopolitics and reimagine regimes of environmental governance (Meilinger and Monstadt, 2021; Savini, 2019). For Savini (2019), the rapid promotion and deployment of CE tactics across some European urban jurisdictions is part of a fundamental shift in the revalorization of waste-as-resource. Put short, circular cities, and their local and regional economies, are driven by waste. In response to the rising challenges of resource costs/insecurities, growing volumes of landfilled waste (despite rising recycling rates), and decreasing industrial productivity, urban policymakers have turned to the CE to ‘redefine urban development as process of generating – not exploiting – resources’ (Savini, 2019: 680). Here, consumers and urban households are recruited and rearticulated as ‘prosumers’ (simultaneous producers and consumers of waste-resources) in an endeavor to develop viable markets for recovered materials. Rather than setting the stage for radical change, however, this new urban economy of materials, made possible through complex practices and networks of recycling, upcycling, but also repairing and sharing, represents a structural adaptation to capitalism in crisis – what some now simply call Capitalism 2.0 (Godelnik, 2021; Hobson and Lynch, 2016). As above, the prevailing CE and its urban correlate, the CC, thus offer a palatable tactic and proclamation of change but do so without seriously engaging with the ‘more fundamental source of ecological problems: ever growing consumer capitalism’ (Savini, 2019: 688).

In sum, as the CE continues to colonize urban (policy) landscapes there is a growing call to critically engage with the ways in which these visions and practices are variously implemented in the processes of urban growth and city making. Moving beyond the approbatory and technocentric ‘aura’ (Keblowski et al., 2020: 145) of recent CE and CC literature is crucial to building more equitable and potentially transformative circularity. And, while scholars have begun to investigate important urban questions in the CE, more research is needed to explore the complex processes and consequences of specific practices recruited in the making of the circular city. With these ideas in mind, the following sections offer a place-based investigation of deconstruction – a circular practice and process increasingly integrated into prevailing visions of the circular city. As we shall see, as a niche but emerging practice, deconstruction is complexly intertwined with local social, economic, political contexts and one that exposes some of the challenges inherent in building the contemporary circular city.

### A circular turn for the built environment: CR&D waste and deconstruction in the circular city

The built material environment has become a central focus of CE proponents and a key target of practitioners and policy makers seeking to build circular cities. This recent turn is perhaps unsurprising given the fact that the material practices of urbanization and continual CR&D cycles are extremely wasteful. While construction productivity has shown signs of slowing in many countries, buildings and infrastructure continue to represent key spaces of structural and energy waste; ranging from growing energy demands and GHG emissions (Dahmen et al. 2018), systematic patterns of building under- and over-utilization, and mounting material and toxic discards (EMF, 2015). Exactly how much waste is generated, however, is a matter of ongoing debate as global estimates range from 1.5 billion tons to 4 billion tons across 40 of the world’s largest economies (Wu et al., 2014; Zheng et al., 2017; Akhtar and Sarmah, 2018). Given their size and rates of growth, China, Europe, and the US represent the global CR&D waste leaders: with Europe producing over 902 million tonnes in 2017; the US generating about 600 million tonnes in 2018; and China reaching 2 billion tonnes annually (Adams et al. 2017; Duan et al. 2019; EPA, 2020). And, of course, CR&D waste is also accumulating across nations outside of these global leaders. In smaller contexts like Canada, estimates for CR&D waste in 2010 peaked around 4 million tonnes (close to one third of total waste generated) (CCME, 2019).

Though these national estimates are important to understanding the scope of the challenge, a focal point of CR&D waste accumulation exists at the urban scale, a situation even more acute in the world’s rapidly growing global and globalizing cities. For instance, in Canada’s largest cities, metropolitan regions like Toronto and Vancouver, steady rates of development have led to surges in building construction, home renovations and demolition (MetroVancouver, 2020). In Vancouver, approximately 3000 buildings are torn down per year, many of which are related to a ‘tear down and replace’ practice linked to decades-long rises in residential real estate values (Dahmen, et al. 2018)^1^.

These and other redevelopment processes have contributed to the production of almost 400,000 tonnes of annual CR&D waste in the metropolitan region (MetroVancouver, 2020).

Given this context, there is a growing volume of research concerning waste management and the CR&D sectors, especially with regards to exploring the role of urban sustainability approaches. Here, the problem of waste and its solutions have been largely framed by technical experts (i.e. engineers, waste management, materials planners) concerned with green building and retrofit technologies, developing clearer waste life-cycle assessments (LCA) (Sharma et al., 2011), and quantifying the environmental consequences of specific waste products (Vieira et al. 2016). Moreover, a dearth of research highlights the value of optimizing waste recycling, reuse and reduction (the 3Rs), but to date much of this work remains limited to exploring the outcomes of recycling specific material streams (i.e. concrete or wood) and tends to ignore a wide range of other contemporary building materials, like insulation or wiring (Silva et al., 2017). Last, while there is growing interest in CR&D waste reduction and prevention (Adams et al. 2017), there remains less comprehensive research concerning reuse practices, policies, and innovations in the CR&D sectors.

Over the last few years, strategies for building circular CR&D sectors have gained considerable momentum. This is made possible, in part, through narratives from leaders like the EMF (2015: 82; 2020) that envision urban managers, planners, and global technology firms as key agents in supporting circular development pathways that ‘produce rather than consume energy and water in liveable urban systems, where circulation and regeneration of resources [are] the norm’. To date, most of the associated CE literature focuses on innovations in construction waste recovery, materials technology, product design, and, in reconfiguring business models in line with innovative service-based strategies. At the forefront of these approaches are technological interventions like 3-D printing and modular (factory-based) construction argued to provide efficiencies in building processes and resource inputs; energy efficient designs like Smart homes and Smart cities that optimize energy performance and virtualize utility delivery; the Platform Economy as a central means for reducing the under/over utilization of urban (residential, office and parking) space; and, more recently, materials recovery through deconstruction. Overwhelmingly, these practices seek to enhance the technological reach of CE and CR&D business-models and deliver ‘end of the pipe solutions’, such as recycling and downcycling (i.e. reducing the value, quality and functionality of the original product) specific CR&D waste. In other words, these ‘best practices’ follow closely with circular interventions in other sectors, like mobility and food, and underscore a central goal of the CE: to ‘construct a paradigm for green economic growth’ based on technological innovation and waste recovery (Savini, 2019: 687).

Given this argument, however, there remains little research that critically explores the integration, application and outcomes of the leading CE tactics in the CR&D sector. I point, in particular, to the growth of deconstruction as one clear and increasingly important practice linked to recent CE and CC initiatives.

### The ‘velvet crowbar’: deconstruction as circular practice

Deconstruction, described by some as ‘construction in reverse’ or the ‘velvet crowbar’, has a long history and has gained favour in recent years as part of an approach in developing ‘green’ building practices (TheReusePeople, n.d.). In contrast to conventional ‘mechanical’ demolition, which often uses large machinery like pulverizers and backhoes that produce mixed piles of debris, deconstruction involves labour intensive practices of hand-by-hand ‘systematic disassembly of a building with the goal of generating a supply of materials suitable for reuse’ (Manuel, 2003: 881).

Limited but sustained research has shown a range of planning and sustainability benefits of deconstruction (McCarthy and Glekas, 2019; Nunes et al. 2019; Paruszkiewicz et al. 2016). Some industry observers, for instance, point to considerable waste reductions with a transition to deconstruction, as some estimates show over 85 percent of demolition discards diverted from landfills and notable reductions in total carbon emissions (McCarthy and Glekas, 2019). In terms of the latter, the carbon benefits from deconstruction are mainly attributed to reductions in the demand for new/raw materials and the sequestration of locked or ‘embodied’ carbon in construction material (i.e. wood) (Sparandara et al. 2019).

While reducing environmental costs, there are indications that deconstruction has direct economic impacts in terms of new job creation, particularly with the need for larger onsite crews, and the potential rise of economic activity in ancillary retail reuse markets (Leigh and Patterson, 2006). Relatedly, McCarthy and Glekas (2019:19; see also Paruszkiewicz et al. 2016) highlight how these economic benefits result in wider social implications: not only do consumers get access to more affordable building materials, but a large proportion of the deconstruction workforce is made up of entry-level positions that employ economically disadvantaged workers.

Even given these benefits, the growth and diffusion of deconstruction, either alongside or as a replacement for traditional demolition, has been limited across most jurisdictions. In the American context, Leigh and Patterson (2006: 219) highlight a number of logistical and institutional barriers, ranging from higher upfront costs of deconstruction over mechanical demolition considering labour and time requirements; the general lack of deconstruction knowledge, skills and expertise across the CR&D industry; and, local policies around the ineligibility of salvageable materials (e.g. reclaimed wood) in construction work (including a number of insurability and liability issues).

Beyond these challenges, the success and growth of the deconstruction sector is also highly contingent on the interest and ability of local experts and the capacity of local salvage markets to circulate recovered materials. In this way, CE leaders have increasingly called for the establishment of more a holistic ‘circular’ mentality that reimagines the source, movement and flow of materials in the CR&D sector. This approach includes supporting a constellation of concepts and practices along a building’s life cycle, including pursuing circular building design (i.e. design for disassembly which supports efficient deconstruction at the end of life) (Adams et al. 2017); material ‘banks’ which reimagine buildings as repositories of quality materials that can be easily recovered; and the development of salvage retail centers or deconstruction hubs to establish a reuse market.

To date there are few examples of comprehensive local or regional deconstruction sectors, let alone deconstruction sectors linked to new CE agendas seeking deeper integration across sectors and spaces. However, as the CE has matured and expanded over the last few years, and as municipalities increasingly support alternative avenues of waste mitigation and reduction, several urban centers have begun to explore the possibilities of deconstruction. In the remainder of this paper, I explore the nascent but developing deconstruction sector in Vancouver and investigate the local strategies and challenges of integrating deconstruction, and material reuse within a wider CE framework. This investigation also illuminates the role of deconstruction in forging a particular vision of the CC, one that reimagines the ‘unbuilt’ environment as a key space for generating waste-resource markets as a means for green growth.

## Methods

This paper is based on twelve semi-structured interviews with key informants involved in local, regional and national waste issues and CE practices (Table 1). Completed from December 2019 to February 2020, these interviews lasted between 40 and 90 minutes and specifically included governmental and non-governmental experts in waste and sustainability policy; municipal planners involved in sustainability and waste strategy; architects; and local demolition/deconstruction experts. The interviews focused on a range of key themes developed from the literature review, including: the existing and emerging challenges concerning CR&D waste; the central policy and planning tools used to mitigate challenges from local and regional waste; the development and integration of CE thinking into current policy and planning initiatives particularly within the built environment sector; and the challenges and opportunities in developing a meaningful local deconstruction sector. The interview data were transcribed, coded, and analyzed qualitatively using NVivo software.

**Table 1.** Characteristics of interview respondents (n = 12).

**Interview**	Type	Date
1	Architect	December 2019
2	Business Improvement Associate, Sustainability Director	December 2019
3	CR&D Expert	December 2019
4	CR&D Expert	January 2019
5	Deconstruction Expert	January 2019
6	Municipal Sustainability Policy Maker	September 2019
7	Municipal Sustainability Policy Maker	January 2020
8	Non-Profit Sustainability/Building Expert	October 2019
9	Non-Profit Sustainability/Building Expert	October 2019
10	Non-Profit Zero Waste/Circular Economy Expert	November 2019
11	Regional Sustainability Policy Maker	February 2020
12	Regional Sustainability Policy Maker	February 2020

This research also integrates data derived from the textual analysis of policy documents associated with CR&D waste planning, CE policy ‘road-maps’, deconstruction practices, and reuse strategies. I explore recent policy and strategy documentation from local agencies including, the Vancouver Economic Board (VEC 2015, 2017, 2020), the City of Vancouver (CoV 2017, 2018), Metro-Vancouver (2020) and, the National Zero Waste Council (NZWC, 2019). Together, these documents are vital to understanding how various stakeholders interpret, integrate and synthesize new ideas and strategies in the waste, sustainability and CE sectors.

### Closing the loop in Vancouver

Much has been made of Vancouver’s rise as a global city. Emerging from its long-established role as the western terminus of the CP rail system and the hub for intensive resource activities (e.g. lumber, mining) in the Pacific Northwest, Vancouver has become a significant post-industrial city (Barnes and Hutton, 2009) and a perennial star in a global showcase of progressive and sustainable urbanism, what Punter (2003) called simply ‘an achievement’. While this achievement is often discussed in terms of urban and community design approaches, the role of environmental activism, sustainability leadership and longstanding public and private engagement in climate change since the 1970s has fundamentally shaped the city’s political landscape (for more see: Affolderbach and Schulz, 2017). To further cement its status, in 2011 the City of Vancouver launched its Greenest City 2020 Action Plan (GCAP), a policy strategy meant to position the city as a global climate change leader. Focused on delivering regional carbon emission reductions and the implementation of renewable energy, the GCAP has also been successful in setting the stage for a comprehensive sustainability policy landscape that includes other key strategies (e.g. Zero Waste 2040; Integrated Solid Waste, and Regional Management Plan); and, meaningful engagements with regional/provincial governments and agencies (e.g. Metro-Vancouver; National Zero Waste Council), and local sustainability leaders (e.g. Light House Sustainable Building Centre).

While this policy and planning landscape has achieved several outcomes, it is also part and parcel of a global urban greening and climate change strategy that critical scholars describe as the ‘sustainability fix’ (Affolderbach and Schulz, 2017). As described above, at question is whether sustainability programs like these result in radical systemic transformations (i.e. strong sustainability) or whether, as is often the case, they represent weaker approaches that result in incremental rather than deep structural change (Bulkeley and Castan Broto, 2012; While et al. 2004). In an increasingly competitive global landscape defined by urban (eco-)entrepreneurialism and innovation, cities like Vancouver are racing to lead the way. Indeed, as Affolderbach and Schulz (2017: 677) argue ‘greening has become not only an environmental but also an economic and political necessity’. Brought forward first as an urban agenda steeped in social development, green and sustainable urbanism is now considered a profitable sector to lead growth and development (Angelo, 2021). For Rosol et al., (2017: 1711), with this urban environmental focus ‘[u]rban actors are not only competing to monetize greenness, they are also increasingly trying to position their city as the “greenest”’.

It is within this evolving context that the CE has emerged in Vancouver not only as a novel sustainability policy direction linked to the Green City strategy and brand, but also as a clear tether to the growing global movement of the CE as a socio-economic model for sustainability action.

To date, the Vancouver region is arguably a Canadian urban leader in experimentations with circularity. While established initiatives like the National Industrial Symbiosis Program (NISP; launched, sited and active in the region) are clear early promoters of CE initiatives, other recent work has focused on delivering circular food systems (CoV, 2017), closed loop textile supply chains (VEC, 2015), neighbourhood revitalization through CE experimentation (i.e. False Creek Flats) (VEC, 2017) and tighter demolition regulations (VEC, 2020).

The last of these projects, formally called the ‘green demolition by-law’, represents a fundamental shift in the practice, process and control of regional CR&D waste. Developed in 2014 as part of the Integrated Solid Waste and Resource Management Plan to divert 80% of regional CR&D waste, the by-law was the first of its kind to establish minimum reuse and recycling requirements for demolition waste from pre-1940 homes. Updates since 2018 have broadened the by-law to include reuse/recycling regulations to homes pre-1950 (with a 75% diversion rate) and all ‘character’ homes pre-1950 (with a 90% diversion rate), and a deconstruction requirement for all heritage listed homes pre-1910 (minimum 3 metric tonnes of wood salvage) (CoV, 2018; see also Teshnizi, 2020). These changes were also accompanied with financial support (up to $250,000) for the establishment and operation of a locally operated non-governmental deconstruction centre or hub.

Overall, the green demolition by-law is an important step in setting the stage for deconstruction and material reuse in the region. And though the regulatory bar is ‘set fairly low… a very small baby step, [it is meant] to create a pipeline of projects that have to be deconstructed’ (Interview 11). Under these conditions, recent estimates put the regional CR&D diversion rates at 78%, with about 0.3 percent of materials put to reuse (VEC, 2020). However, as deconstruction practices evolve and expand across the region (Table 2), recent reports claim that even a 10 percent salvage rate would lead to over 179,078 tonnes of reusable materials annually (VEC, 2020). Clearly, estimates like these are largely based not only on growth of this niche industry, but also on the development of a functional material hub (or series of hubs) that formalize salvage markets, and that build wider acceptance of the deconstruction practice.

**Table 2.** The number of deconstruction and salvage services by location in Vancouver’s Metropolitan Region (total number of service providers = 29) (adapted from: MetroVancouver, 2020: 24-27).

**Municipality**	Deconstruction Appraiser	Deconstruction Services	Salvage Services	Used Materials Store
Burnaby		2	2	1
Delta			1	1
Langley	1			2
Maple Ridge		3	2	3
North Vancouver				2
Port Coquitlam				1
Richmond	1	2	2	
Surrey		2		2
Vancouver		4	5	4
**Total Services Available**	2	13	12	16

### Unbuilding the ‘demolition capital of the world'

In a recent episode of Dragon’s Den, a popular reality show where budding entrepreneurs pitch their businesses to wealthy investors (referred to as ‘Dragons’), a participant, Adam Corneil, founder and CEO of the Unbuilders, declares that ‘Vancouver is the demolition capital of the world’ (Interisano, 2020). Digging into his pitch, Corneil explains that with an estimated ‘20,000 homes slated for demolition in the next 20 years’, Unbuilders, regarded by many as Canada’s only dedicated deconstruction company, is uniquely positioned to intervene in this expanding and increasingly lucrative market. As he puts it, reclaimed lumber and other important construction materials, are ‘not waste, they are just wasted’ (Interisano, 2020).

Echoing Corneil’s sentiment, all of the participants and stakeholders interviewed for this research reflected on the economic potential of deconstruction and many also argued for a number of other tangible and intangible benefits especially as a means of meeting an emerging regional CE. Most notably, several interviewees highlighted the role of deconstruction as a clear pathway to closing valuable material loops. While materials like windows, doors and interior fixtures are important salvage, scarce wood, including old growth lumber and flooring (e.g. fir and oak), represent the mainstay of a deconstruction/reuse market (Teshnizi, 2020). Here, the financial value of old growth wood is widely regarded as a key element with estimated retail prices at five times those of modern lumber (Interisano, 2020). This scarce resource, however, represents more than retail profit as interviewees highlighted its environmental value (e.g. carbon sequestration), its aesthetic quality, and, importantly, its pivotal role in supporting material heritage:

We live in relatively young cities, especially on the West Coast. Vancouver was founded in 1886 and it was built with ancient trees… the wood behind the walls holding up our city is over 500 years old, sometimes a thousand years old! That is the heritage of this land. And it needs to be seen in that respect. It’s not just an infinite wood resource that we can go in and cut down and build with it. It’s finite. And every demolition that happened makes it more and more scarce. (Interview 5)

Similarly, another interviewee highlighted the role of this heritage wood as a fundamental part of the story of Vancouver, a material legacy, that challenges the modern process of rapid teardown and replacement that is transforming the city:

The public views the loss of the older homes, the character homes as part of the loss of Vancouver’s heritage. Even just the removal of those buildings is a sensitive topic for the public. I think everybody has got it, when you see these buildings just smashed by a backhoe and hauled off and then is splintered fragments. It bothers everybody. From a heritage perspective, I think we’re starting to think of it more like that. The wood … it’s old growth and that’s part of the story of this place. And saving the wood and reusing then in decorative visible ways is part of keeping that story local… and it’s part of our legacy as Canadians. (Interview 6)

The value of deconstruction in maintaining local, regional, and indeed, national, heritage is gaining momentum. Heritage and architectural scholars, for instance, are paying increasing attention to the role of waste, deconstruction, and reuse not only in specific articulations of conservation studies but also in the ways that these elements challenge traditional definitions of heritage (Ross, 2020). Indeed, the rise of deconstruction in Vancouver, as elsewhere, offers a direct way to engage in what McCarthy and Glekas (2019: 16) describe as the heritage goals of preserving memory while supporting a sense of ‘revival’ and sustainable community development.

And, yet even given these, and other, benefits, the lack of awareness of deconstruction as a viable (and marketable) intervention in the Canadian CR&D sector results in number of key obstacles. Across the interviews, many highlighted persistent local and regional challenges to building a comprehensive deconstruction market.

The first challenge, and one that directly reflects the existing literature (Manuel 2003; Leigh and Patterson, 2006), concerns the financial and time costs in contrast to mechanical demolition. All of the interviewees highlighted ongoing issues with overcoming both the real and perceived costs associated with deconstruction contracts. By and large, deconstruction firms and their clients must consider the up-front costs associated with this more complex process which typically moves through several, potentially lengthy, phases: auditing, inspecting, and planning; deconstruction and material separation; and post-deconstruction material processing (Interview 10).

In the first phase, most deconstruction projects require site inspections and material audits. Key to this process is the fact that deconstruction markets rely on returns (i.e. tax receipts) from charitable donations; revenue that is ‘recovered at the end of the process’ (Interview 10). In some cases, a deconstruction appraiser, a niche professional different from the typical home appraiser, determines what materials can be salvaged, estimates the value of the donations, and prepares a report, including tax documentation, that lists the donation and market value (Jordan, 2016). For the Unbuilders, some of these costs are reduced through free ‘salvage audits’ (Interview 5) and more recently through technological interventions, like the use of specialized crane systems, to speed up the last two phases (Johnston, 2020). Overall, then, a key issue in supporting deconstruction over traditional demolition remains finding ways not only to reduce upfront costs (of time and money) but to also to promote the net financial and material benefits at the end of the process.

A second challenge involves the shortage of deconstruction knowledge, skills, and expertise in the local and regional CR&D labour markets. As might be expected considering the lack of awareness in Vancouver, and across Canada, there remains little institutional capacity among vocational colleges and polytechnic institutes (e.g. British Columbia Institute of Technology, BCIT) to develop comprehensive deconstruction programs. While institutions like BCIT, arguably the region’s largest educator for CR&D training, are showing clear interest in sustainability and the CE (Chan, 2019), there remains a limited demand for deconstruction skills, including techniques around salvage and material reuse, in the building sector. As one local policy expert highlighted,

the problem is that [BCIT] looked at deconstruction and it fits nicely with the direction they’re trying to go, but the number of people they think would be interested in taking that course is so small, so niche that it just didn't seem worth it to go through all the work to create a course curriculum and all that investment … [It’s] just not possible in the short term, maybe in the long term as we transition all ‘demo’ companies and then phase out and everything’s deconstruction. But right now, maybe one or two companies might send a couple guys. So, you can’t create a whole course. (Interview 7)

While a full-scale transition to deconstruction is a longer-term goal, especially as the City has only recently increased green demolition requirements and continues to experiment with circular thinking, others point to short term alternatives like ‘on-site training and speciality apprenticeships’ to close the gaps (Interview 8). According to a practitioner, ‘having your first-year carpenters do a few days of deconstruction, I think would be great. We have a few carpenters’ apprentices working with our company that are new that are going through their tickets. I think it’s really valuable’ (Interview 8). But of course, deconstruction skills should not be merely relegated to niche apprenticeships. As one interviewee described, broadening the knowledge of deconstruction to other tradespeople and indeed cultivating a wider sensitivity to the value of circular thinking and salvageable building materials is an important step:

a big part of it goes to educating the other trades … an [uninformed] crew can easily come in and destroy all of the wood and just do really silly things, like destroy all the windows. We have to educate the other subtrades that are involved in a circular building removal process (Interview 11).

Third, several participants highlighted a growing concern about the direction that local waste companies are taking in terms of appropriating deconstruction as a core service yet without following established deconstruction principals. In other words, there is a perceived gap between ‘legitimate’ deconstruction professionals, few as they are, and those that are deploying deconstruction as merely a branding exercise – what one interviewee labelled ‘greenwashing’:

over the last five years it is just crazy how badly deconstruction has become greenwashed … you have contractors, demolition companies here saying that they do deconstruction. They all have a tab on their website that says ‘deconstruction’. But they're actually doing ‘building recycling’. They still smash [buildings] down with the machines, they just don't take the material to a landfill. They take it to a chipper, and they call that deconstruction! (Interview 12)

In this case, differentiating between recycling and reusing is much more than mere semantics. Indeed, a key aspect of meaningful circularity concerns re-defining the value of materials and re-organizing the ‘loops’ through which our discards travel. Though some materials certainly lend better to recycling streams (e.g. metals), the practices of refurbishment, repair and direct reuse, fundamental aspects of deconstruction, are part and parcel of a process of retaining value and extending the useful life of materials. The argument, in short, is that this mislabelling of recycling as deconstruction threatens not only deconstruction as a brand but potentially undermines ‘the very project of a radical way of doing the Circular Economy. This “circular washing” challenges what we are working toward’ (Interview 10). In response, one interviewee described the role of legitimate deconstruction practitioners in taking on key leadership roles in terms of developing industry knowledge, that is, by intervening through on-the-ground explanations with other CR&D practitioners:

My focus is with the builders, developers and architects to tell them, like, ‘you got to know what you’re hiring. You’re not hiring a deconstruction company… You are hiring a demolition company that's telling you they’re doing something that they are not’. (Interview 5)

Along these lines, several participants described the need to develop, disseminate, and eventually, enforce industry standards. Much of these tasks have, for the moment, fallen to non-profit organizations and invested firms like the Unbuilders. One social enterprise, for instance, is seeking funding to develop a locally specific ‘design for disassembly’ guideline (a manual that outlines best practices for engaging experts in innovative design strategies that support circularity from the very beginning). Similarly, a local practitioner explained how his firm is ‘trying to build a useful blueprint so that other contractors can shift over and start doing [deconstruction]. We're not just doing this for ourselves to grow a big company… Our mission is to make deconstruction the industry standard’ (Interview 5).

A final, but increasingly pivotal aspect of forging a viable deconstruction and circular CR&D economy concerns the development and support of a deconstruction commons – a hub or centre to house and trade deconstruction salvage to local and regional markets. At present, the retail environment for CR&D materials is expanding with 16 used material stores across the region (Table 2). For most practitioners, the Habitat for Humanity ReStore program, a charitable salvage retailer, represents a crucial space for maintaining the deconstruction sector and the central source of financial returns (i.e. tax receipts) for deconstruction projects. While important, however, the types of salvage and the size of loads that these and other sites offer is remarkably variable, and most significantly, are part of a decentralized and loosely organized materials recovery system. As one interviewee put it,

[demolition and deconstruction firms] don’t want to go to 10 different places to drop stuff off. They want to go to one place and move it… we have five or six materials to come off of every site. And let’s go to one place and put all the glass on one side, put the wood down the other side, and have metal and concrete in the middle of the court… that is the ideal scenario (Interview 3).

In response, the City of Vancouver (2018) has committed financial support (up to $250,000 from the Innovation Fund) and continues to explore the establishment of an independently operated and public-facing hub meant to facilitate and encourage building material reuse market. As a non-governmental space coordinated though public and private collaboration, the hub is expected to be managed and to evolve through the input of local experts, industry practitioners and community stakeholders. Though the program has gained some traction in the last few years, there does remain clear challenges in coordinating such a diverse range of local stakeholders and in sustaining long term engagement. In the words of regional policy expert, ‘what we were thinking 10 years ago was that: build it [a deconstruction hub] and they [deconstruction experts] will come, and all your problems will be solved… not so’ (Interview 12). Along these lines a practitioner argued that,

I think to get too many parties involved is going to be a disaster. In my opinion, I've been in these conversations for four years now and all this conversation is hard to have… we need just three people at the table so we can actually get something done… everyone is interested and talking about it but no one wants to put their money into it (Interview 4).

While engagement and collaboration issues continue, another clear challenge yet to be resolved concerns the location of a hub. Several interviewees highlighted the reality that real-estate costs preclude the opportunity to site the hub in or close to Vancouver’s urban core, the space with the most potential for deconstruction and the flow of salvage materials. As project champions ‘come to terms with this prospect’ (Interview 12), others point out that regardless of where the hub ends up, it must represent more than merely a space of material reuse. In this case, most interviewees reflected on the wider value of a deconstruction hub as a space of material *and* knowledge/cultural exchange. While the hub will clearly facilitate the exchange of salvaged materials (e.g. inspection, standardization, remanufacture and sale), its role as a centre for knowledge and cultural exchange is paramount (Interview 1) (see also, Teshnizi, 2020). As an architectural commons, the hub has the potential to offer skills training for both CR&D practitioners and the public, while acting as a regional platform to showcase innovations in sustainability and green building, and as one interviewee put it, ‘a space to experiment with new ideas in the Circular Economy’ (Interview 1).

## Concluding reflections

On a planet of finite resources, the circular economy is not optional, it is inevitable.

∼ Hermann Erdmann, CEO, REDISA (EMF, 2015: 8)

Few sustainability initiatives have gained as much attention as the CE over the last several years. For some, this ‘inevitable’ agenda lies in its capacity to capitalize on established sustainability frameworks and to harness the technological revolution, both of which have found remarkable purchase in the urban context. As urban regions become the main targets in achieving global sustainability, cities are increasingly at the center of efforts to apply and test circular economy initiatives and policy approaches. While experiments in the key sectors like textiles, transportation, and food have driven much of the attention of late, new initiatives and policy directions are being aimed at the built environment and the intensifying waste generated by the CR&D sectors.

Though deconstruction has existed as a viable practice in the construction/demolition sector for years, it has generated remarkably little attention by both industry leaders and scholars. That is until more recently. With the global popularity of circular talk and the rise of innovative systems to meet sustainability and resource demands, alternative practices like deconstruction are gaining momentum especially in the endeavor to build circular cities (EMF, 2017; VEC, 2020). And yet, outside of key contexts like Western Europe, deconstruction is still regarded as an unorthodox practice. Even in a city as progressive and environmentally focused as Vancouver, deconstruction is largely viewed as a technique and approach that ‘flies under the radar’ in an industry that is responsible for a substantial and growing amount of waste (Interview 5).

Overall, the existing literature, while vital, offers little explanation of the dynamic challenges, incremental successes, and socio-environmental impacts of the deconstruction sector in North America, let alone Canada, and its increasing ties to emerging CE and CC agendas. This paper responds to this narrow focus by highlighting the recent role of deconstruction in closing CR&D loops and establishing a space for the revalorisation of building waste-as-resource in Vancouver. While contributing to scholarship on the CE and deconstruction, and their impacts on society and industry, this investigation presents several implications worth outlining.

First, as urban leaders embrace the CE agenda and seek to remake and rebrand their cities, there is an increasing need to engage with the existing practices that make up this circular turn. Indeed, while deconstruction represents a viable, though fledgling opportunity space to reconfigure CR&D waste, in line with recent critical literature this research suggests that this practice is also embedded in a wider project of green growth. In Vancouver, deconstruction is now being recruited as a lever of the green demolition policy, a policy that is part and parcel of a particular metropolitan vision of circularity and the next step in creating the circular city. But of course, the promotion of deconstruction as a viable, sustainable and, indeed circular, alternative requires considerable support and investment from a variety of stakeholders. The knowledge, experiences and interpretations of local experts in Vancouver, from practitioners to policy makers, highlight the complex challenges of building momentum for deconstruction, either as a legitimate practice alongside conventional demolition, or as one that has the potential to replace it. Here, increased funding from local, regional, and indeed, national, governments is considered as one key step. Several participants, for instance, called for more funding through local grants to support both the development of deconstruction businesses and to test new approaches in local markets. So too, support for the development of guidelines (regional and national) of the CE and deconstruction, including issues of enforcement, were highlighted as key steps. Furthermore, with public sector support, educational institutions also have an increasingly important role to play. In Vancouver, the shortage of trained workforce and skills in deconstruction is starting to show and are clear obstacles not only for employers seeking to expand deconstruction services, local and regionally, but also for the industry *writ large.* Overall, these issues point to a prioritization of political, economic, and technical investments as key interventions in building urban circularity, investments which side-step complex questions of how deconstruction might play in presenting palatable as opposed to radical change in a rapidly growing city.

Additionally, while the City of Vancouver has earmarked substantial financial support for a deconstruction hub, its success is much more than simply building a facility. Indeed, an architectural or deconstruction commons will require the development, facilitation, and maintenance of salvage networks – both in a physical and a social sense. On the one hand, establishing a regional CE of CR&D waste is contingent on local political and economic contexts; in this case, one that is deeply implicated in an overheated real estate market. On the other hand, the continual flow and re-valuation of salvage material relies on building robust social networks, interactions and relationships between buyers, sellers, and other agents. This then represents a challenging context that requires champions (at all levels) to invest and commit to deconstruction in the long term.

Second, with deepening ties to emerging CE agendas, there is a need to explore how deconstruction, as a practice and a culture, is shaped by the complex social, economic and political nature of the CC, and vice versa. What benefits and challenges exist for deconstruction as the practice is increasingly understood as part and parcel of the CE? As the CE picks up steam, especially across Canadian cities, there are questions about how new and existing actors/agents shift their practices and processes to meet new closed-loop standards and expectations. Though talk of the CE is gaining traction in Canada, it largely remains a concept and agenda with little standards or even resonance especially outside of waste sectors. Notable here is the rise of green, or more aptly, circle-washing by local practitioners seeking to plug into the loose rhetoric of deconstruction and circularity, allegedly without the knowledge or experience to do so. While appropriating circular language on websites and brochures might seem of little consequence, such acts are likely to erode the value of the CE and threaten trust throughout the industry, especially if practitioners eschew the emerging salvage and reuse markets in favour of cheaper and easier recycling and landfilling streams.

A final and central issue concerns not merely how we apply CE thinking to the built environment, but perhaps more importantly, how we understand role of the CE as a wider social, cultural and economic intervention. While the prevailing technological and material foci of the CE and deconstruction allow us to envision new resource efficiencies, we also need to understand their roles in legitimizing new accumulation regimes that envision the discards of our built material environments as key inputs for future growth (Savini, 2019). More research is thus required to understand how deconstruction can work alongside new or alternative paradigms of city making that envision meaningful change. In the end, as this work has attempted to underscore, a transformative CE for the bult environment in Vancouver and beyond means as much about how we approach *un*building our cities as it does building them.

## Notes

1. According to Dahmen et al. (2018: 96), in the past 30 years, ‘26,800 single family homes, or 40% of all single-family homes in residentially (RS) zoned areas, were demolished and replaced with new single family homes’.

## References

[bibr1-0308518X221116891] Abramson D (2016) Obsolescence: An Architectural History. Chicago: The University of Chicago Press.

[bibr2-0308518X221116891] Adams KT Osmani M Thorpe T , et al. (2017) Circular economy in construction: Current awareness, challenges and enablers. Proceedings of the Institution of Civil Engineers-Waste and Resource Management. 15–24.

[bibr3-0308518X221116891] Affolderbach J Schulz C (2017) Positioning Vancouver through urban sustainability strategies? The Greenest City 2020 Action Plan. Journal of Cleaner Production 164: 676–685.

[bibr4-0308518X221116891] Akers J Béal V Rousseau M (2020) Redefining the city and demolishing the rest: the techno-green fix in postcrash Cleveland, Ohio. Environment and Planning E: Nature and Space 3(1): 207–227.

[bibr5-0308518X221116891] Akhtar A Sarmah A (2018) Construction and demolition waste generation and properties of recycled aggregate concrete: A global perspective. Journal of Cleaner Production 186: 262–281.

[bibr6-0308518X221116891] Angelo H (2021) How Green Became Good: Urbanized Nature and the Making of Cities and Citizens. Chicago, IL: University of Chicago Press.

[bibr7-0308518X221116891] Barnes T Hutton T (2009) Situating the new economy: Contingencies of regeneration and dislocation in Vancouver's inner city. Urban Studies 46(5-6): 1247–1269.

[bibr8-0308518X221116891] Bassens D Kębłowski W Lambert D (2020) Placing cities in the circular economy: Neoliberal urbanism or spaces of socio-ecological transition? Urban Geography 41(6): 893–897.

[bibr9-0308518X221116891] Bulkeley H Castán Broto V (2012) Government by experiment? Global cities and the governing of climate change. Transactions of the Institute of British Geographers 38(3): 361–375.

[bibr10-0308518X221116891] Cairns S Jacobs JM (2014) Buildings Must Die. Cambridge, Mass.: MIT Press.

[bibr11-0308518X221116891] Canadian Council of Ministers of the Environment (CCME) (2019) Guide for identifying, evaluating and selecting policies for influencing construction, renovation, and demolition waste management. CCME: Winnipeg, online: https://www.ccme.ca/files/Resources/waste/wst_mgmt/CRD%20Guidance%20-%20secured.pdf.

[bibr12-0308518X221116891] Chan C (2019) Two BCIT students are cultivating the circular and repair economy in Metro Vancouver, BCIT News, online: commons.bcit.ca/news/2019/07/right-to-repair-metro-vancouver/.

[bibr13-0308518X221116891] Cidell J (2009) Building green: The emerging geography of LEED-certified buildings and professionals. The Professional Geographer 61(2): 200–215.

[bibr14-0308518X221116891] City of Vancouver (CoV) (2017) Vancouver Food Strategy Progress Report and Action Plan Update, Council Committee Report: RTS #11893, online: //vancouver.ca/files/cov/final-food-strategy-report-back-and-update-2017-rts-11893.pdf.

[bibr15-0308518X221116891] City of Vancouver (CoV) (2018) Green Demolition by-law update: RTS #12213, online: //council.vancouver.ca/20180516/documents/pspc2c.pdf.

[bibr16-0308518X221116891] Creba A Devlieger L (2019) Deconstructing research: A reverse–engineering methodology and practice. Architectural Design 89(3): 96–101.

[bibr17-0308518X221116891] Dahmen J von Bergmann J Das M (2018) Teardown index: Impact of property values on carbon dioxide emissions of single-family housing in Vancouver. Energy and Buildings 170: 95–106.

[bibr18-0308518X221116891] Duan H Miller TR Liu G , et al. (2019) Construction debris becomes growing concern of growing cities. Waste Management 83: 1–5. 30514455 10.1016/j.wasman.2018.10.044

[bibr19-0308518X221116891] Edensor T (2005) Waste matter: The debris of industrial ruins and the disordering of the material world. Journal of Material Culture 10(3): 311–332.

[bibr20-0308518X221116891] Ellen MacArthur Foundation (2015) Growth Within: A Circular Economy Vision for a competitive Europe, online: www.ellenmacarthurfoundation.org/assets/downloads/publications/EllenMacArthurFoundation_Growth-Within_July15.pdf.

[bibr21-0308518X221116891] Ellen MacArthur Foundation (2017) Cities in the Circular Economy: An Initial Exploration, online: www.ellenmacarthurfoundation.org/assets/downloads/publications/Cities-in-the-CE An-Initial-Exploration.pdf.

[bibr22-0308518X221116891] Ellen MacArthur Foundation. (2020) Circular Economy in Cities: An Initial Exploration, online: www.ellenmacarthurfoundation.org/our-work/activities/circular-economy-in-cities.

[bibr23-0308518X221116891] Environmental Protection Agency (EPA) (2020) Advancing Sustainable Materials: 2018 Fact Sheet. EPA: Office of Land and Emergency Management, 530-F-20-007, online: https://www.epa.gov/sites/production/files/2020-11/documents/2018_ff_fact_sheet.pdf.

[bibr24-0308518X221116891] Godelnik R (2021) The rise of (Mc)Circular economy. In: Rethinking Corporate Sustainability in the Era of Climate Crisis. Cham: Palgrave Macmilian, pp.67–80.

[bibr25-0308518X221116891] Gregson N Crang M Fuller S , et al. (2015) Interrogating the circular economy: The moral economy of resource recovery in the EU. Economy and Society 44(2): 218–243.

[bibr26-0308518X221116891] Hobson K (2016) Closing the loop or squaring the circle? Locating generative spaces for the circular economy. Progress in Human Geography 40(1): 88–104.

[bibr27-0308518X221116891] Hobson K (2020) From circular consumers to carriers of (unsustainable) practices: Socio-spatial transformations in the circular city. Urban Geography 41(6): 907–910.

[bibr28-0308518X221116891] Hobson K Lynch N (2016) Diversifying and de-growing the circular economy: radical social transformation in a resource-scarce world. Futures 82: 15–25.

[bibr29-0308518X221116891] Interisano J (2020) no title. [Season 15, Episode 2]. In Tracie Tighe, Molly Middleton, Alexandra Lane, Amy Bourne (Executive Producers), Dragon’s Den, CBC/Radio-Canada.

[bibr30-0308518X221116891] Johnston J (2020) Vancouver crew ‘unbuilds’ home in record time, as it aims to offset demolition waste. CBC News, Oct 1, online: www.cbc.ca/news/canada/british-columbia/unbuilders-home-deconstruction-1.5745605.

[bibr31-0308518X221116891] Jordan W (2016) Deconstruction can be a tax-savvy alternative to demolition. The Washington Post, Aug 25. Online: www.washingtonpostcom/realestate/deconstruction-can-provide-huge-tax-benefits-for-property-owners/2016/08/24/8f6c5270-62fb-11e6-96c0-37533479f3f5_story.html.

[bibr32-0308518X221116891] Kębłowski W Lambert D Bassens D (2020) Circular economy and the city: An urban political economy agenda. Culture and Organization 26(2): 142–158.

[bibr33-0308518X221116891] Kirchherr J Reike D Hekkert M (2017) Conceptualizing the circular economy: An analysis of 114 definitions. Resources, Conservation and Recycling 127: 221–232.

[bibr34-0308518X221116891] Leigh NG Patterson L (2006) Deconstructing to redevelop: A sustainable alternative to mechanical demolition. Journal of the American Planning Association 72(2): 217–225.

[bibr35-0308518X221116891] Lynch N (2022) Remaking the obsolete: Critical geographies of contemporary adaptive reuse. Geography Compass 16(1): e12605.

[bibr36-0308518X221116891] Manuel J (2003) Unbuilding for the environment. Environmental Health Perspectives 111(16): A880–A887. 14644683 10.1289/ehp.111-a880 PMC1241780

[bibr37-0308518X221116891] McCarthy TM Glekas E (2019) Deconstructing heritage: Enabling a dynamic materials practice. Journal of Cultural Heritage Management and Sustainable Development 10(1): 16–28.

[bibr38-0308518X221116891] Meilinger V Monstadt J (2021) From the sanitary city to the circular city? Technopolitics of wastewater restructuring in Los Angeles, California. International Journal of Urban and Regional Research 46(2): 182–201.

[bibr39-0308518X221116891] MetroVancouver, (2020) Construction and Demolition: Waste reduction and recycling toolkit. A guide for the building and construction industry. October, online: www.metrovancouver.org/services/solid-waste/SolidWastePublications/DLCToolkit.pdf.

[bibr40-0308518X221116891] Mylan J Holmes H Paddock J (2016) Re-Introducing consumption to the ‘circular economy’: A sociotechnical analysis of domestic food provisioning. Sustainability 8: 794.

[bibr41-0308518X221116891] Nunes A Palmeri J Love S (2019) Deconstruction vs. Demolition: An Evaluation of Carbon and Energy Impacts from Deconstructed Homes in the City of Portland. Portland, OR: State of Oregon Department of Environmental Quality.

[bibr4200-0308518X221116891] NZWC (2019) Circular Cities: A Scan of Global Approaches and Key Takeaways for Canadian Local Governments, May, online: www.nzwc.ca/Documents/CircularCitiesScanSummaryReport.pdf.

[bibr42-0308518X221116891] Paruszkiewicz M Liu JH Hanes R , et al. (2016) The Economics of Residential Building Deconstruction in Portland, OR.

[bibr43-0308518X221116891] Peck J Theodore N (2015) Fast Policy: Experimental Statecraft at the Thresholds of Neoliberalism. Minneapolis, MN: The University of Minnesota Press.

[bibr44-0308518X221116891] Prendeville S Cherim E Bocken N (2018) Circular cities: Mapping six cities in transition. Environmental Innovation and Societal Transitions 26: 171–194.

[bibr45-0308518X221116891] Punter J (2003) The Vancouver Achievement: Urban Planning and Design. Vancouver: UBC Press.

[bibr46-0308518X221116891] TheReusePeople (n.d.) The Velvet Crowbar, online: www.thereusepeople.org/velvetcrowbar.

[bibr47-0308518X221116891] Rosol M Béal V Mössner S (2017) Greenest cities? The (post-)politics of new urban environmental regimes. Environment and Planning A: Economy and Space 49(8): 1710–1718.

[bibr48-0308518X221116891] Ross S (2020) Re-evaluating heritage waste: sustaining material values through deconstruction and reuse. The Historic Environment: Policy & Practice 11(2-3): 382–408.

[bibr49-0308518X221116891] Savini F (2019) The economy that runs on waste: Accumulation in the circular city. Journal of Environmental Policy & Planning 21(6): 675–691.

[bibr50-0308518X221116891] Sharma A Saxena A Sethi M , et al. (2011) Life cycle assessment of buildings: A review. Renewable and Sustainable Energy Reviews 15(1): 871–875.

[bibr51-0308518X221116891] Silva A Rosano M Stocker L , et al. (2017) From waste to sustainable materials management: Three case studies of the transition journey. Waste Management 61: 547–557. 27955907 10.1016/j.wasman.2016.11.038

[bibr52-0308518X221116891] Sparandara L Werner M Kaminsky A , et al. (2019) Accelerating the Circular Economy through commercial deconstruction and reuse. [White paper]. Ellen Macarthur Foundation & Google. https://www.ellenmacarthurfoundation.org/assets/downloads/google-deconstruction-and-reuse.pdf.

[bibr53-0308518X221116891] Teshnizi Z (2020) Vancouver pre-1940 houses: A cache for old-growth forest wood. Journal of Cultural Heritage Management and Sustainable Development 10(1): 41–51.

[bibr54-0308518X221116891] Valenzuela F Böhm S (2017) Against wasted politics: A critique of the circular economy. Ephemera 17(1): 23–60.

[bibr55-0308518X221116891] Vancouver Economic Commission (VEC) (2015) Towards the Circular Economy: Identifying local and regional government policies for developing a circular economy in the fashion and textiles sector in Vancouver, Canada, online: www.vancouvereconomic.com/research/towards-the-circular-economy/.

[bibr56-0308518X221116891] Vancouver Economic Commission (VEC) (2017) The Flats: Industry, Sustainability, and Innovation, online: www.vancouvereconomic.com/wp-content/uploads/2017/05/Flats-Strategy_Final_May8.compressed-1.pdf.

[bibr57-0308518X221116891] Vancouver Economic Commission (VEC) (2020) The Business Case for Deconstruction: Economic and environmental impacts of a demolition-deconstruction shift in Metro Vancouver, Canada, online: www.vancouvereconomic.com/research/the-business-case-for-deconstruction/.

[bibr58-0308518X221116891] Vieira DR Calmon JL Coelho FZ (2016) Life cycle assessment (LCA) applied to the manufacturing of common and ecological concrete: A review. Construction and Building Materials 124: 656–666.

[bibr59-0308518X221116891] WEF (World Economic Forum) (2018) Circular economy in cities: Evolving the model for a sustainable urban future. http://www3.weforum.org/docs/White_paper_Circular_Economy_in_Cities_report_2018.pdf.

[bibr60-0308518X221116891] While A Jonas AEG Gibbs D (2004) The environment and the entrepreneurial city: searching for the urban ‘sustainability fix’ in Manchester and Leeds. International Journal of Urban and Regional Research 28(3): 549–569.

[bibr61-0308518X221116891] Williams J (2021) Circular Cities: A Revolution in Urban Sustainability. London: Routledge.

[bibr62-0308518X221116891] Wu Z Ann TW Shen L , et al. (2014) Quantifying construction and demolition waste: An analytical review. Waste Management 34(9): 1683–1692. 24970618 10.1016/j.wasman.2014.05.010

[bibr63-0308518X221116891] Zheng L. Wu H. Zhang H ., et al. (2017). Characterizing the generation and flows of construction and demolition waste in China. Construction and Building Materials, 136, 405–413.

